# Supplementary material for: Efficacy and Safety of Bifidobacterium longum Supplementation in Infants: A Meta-Analysis of Randomized Controlled Trials
Source: Foods. 2023 Dec 12;12(24):4451. doi: 10.3390/foods12244451 (PMC10742581; doi:10.3390/foods12244451)
Supplement: Supplementary file 1 [file foods-12-04451-s001.zip › foods-2692026-supplementary.pdf]

Supplementary Material

Efficacy and Safety of Bifidobacterium Longum Supplementation in Infants: A Meta-analysis of Randomized Controlled Trials

Huangda Guo, BS[1], Meng Fan, MS[1], Tianjiao Hou, BS[1], Yixin Li, BS[1], Siyue Wang, MPH[1], Xueheng Wang[1], Hexiang Peng, MS[1], Mengying Wang, PhD[3], Tao Wu, PhD[1, 2, *], Yumei Zhang, PhD[3, *]

*** Correspondence:**
Tao Wu, Department of Epidemiology and Biostatistics, School of Public Health, Peking University, Beijing, 100191, China. E-mail: twu@bjmu.edu.cn

Yumei Zhang, Department of Nutrition and Food Hygiene, School of Public Health, Peking University, Beijing, 100191, China. E-mail: zhangyumei@bjmu.edu.cn

1 Supplementary Data

None.

2 Supplementary Figures and Tables

Table S1 Detailed literature search information

Databases	Search terms
PubMed	(Bifidobacterium longum [MeSH Terms] OR Bifidobacteri* OR BlSP * OR B. longum OR B. longum subsp. longum) OR (Bifidobacterium longum subspecies infantis [MeSH Terms] OR Bifidobacterium infantis OR Bifidobacterium longum subsp. Infantis OR B. infantis OR Bifidobacterium longum subsp. infantis OR Bifidobacterium longum ssp. infantis OR B. longum subsp. infantis)AND ((Infant* OR Infant, Newborn [MeSH Terms]OR newborn OR neonat*) OR (Child* OR Child, Preschool [MeSH Terms] OR children* OR young* OR toddler))AND (Randomized Controlled Trial* OR Controlled Clinical Trial* OR Trials)
EMBASE	Bifidobacterium longum'/exp OR ' Bifidobacterium*' OR ‘BlSP*’ OR ‘B. longum’ OR ‘B. longum subsp. Longum’ OR (‘Bifidobacterium longum subspecies infantis’/exp OR ‘Bifidobacterium infantis’ OR ‘Bifidobacterium longum subsp. Infantis’ OR ‘B. infantis OR Bifidobacterium longum subsp. Infantis’ OR ‘Bifidobacterium longum ssp. Infantis’ OR ‘B. longum subsp. Infantis’) AND ((‘Infant’ /exp OR ‘Newborn’ OR ‘neonat*’) OR (‘Child’/exp OR ‘Preschool’ OR ‘Child*’ OR ‘young*’ OR ‘toddler’))AND (‘Randomized Controlled Trial’/exp OR ‘Controlled Clinical Trial’ OR ‘Trial’)

Cochrane Library	(Bifidobacterium longum [MeSH Terms] OR Bifidobacteri* OR BlSP * OR B. longum OR B. longum subsp. longum) OR (Bifidobacterium longum subspecies infantis [MeSH Terms] OR Bifidobacterium infantis OR Bifidobacterium longum subsp. Infantis OR B. infantis OR Bifidobacterium longum subsp. infantis OR Bifidobacterium longum ssp. infantis OR B. longum subsp. infantis)AND ((Infant* OR Infant, Newborn [MeSH Terms]OR newborn OR neonat*) OR (Child* OR Child, Preschool [MeSH Terms] OR children* OR young* OR toddler))AND (Randomized Controlled Trial* OR Controlled Clinical Trial* OR Trials)
SinoMed	("随机对照试验"[全部字段:智能] OR "临床试验"[全部字段:智能] OR "试验"[全部字段:智能]) AND ("婴儿"[全部字段:智能] OR "婴幼儿"[全部字段:智能] OR "幼儿"[全部字段:智能] OR "学龄前"[全部字段:智能] OR "儿童"[全部字段:智能]) AND ("长双歧杆菌"[全部字段:智能] OR "长双歧杆菌长亚种"[全部字段:智能] OR "长双歧杆菌婴儿亚种"[全部字段:智能])

Table S2 Detailed description of basic characteristics of the studies included in the meta-analysis

Study	Publication year	Country	Patients[1]	Female	Birth weight (g)	Gestational age (week)	Cesarean[1]	Postnatal age at starting (month)	Probiotic strains	dose (CFU/d)	Duration of supplementation	Antibiotic exposure	Type of milk feeding	Outcomes[2]
Al-Hosni et al.(6)	2012	America	50/51	49.50%	778	26	22/30	4	Bifidobacterium longum infantis, Lactobacillus rhamnosus GG	5×10^8	34 weeks	no	breast milk	a
Mark et al.(20)	2006	America	31/29	36.70%	1428	30	23/23	7	Bifidobacterium longum, Bifidobacterium longum infantis, Lactobacillus acidophilus, Bifidobacterium bifidum	5×10^8	5 weeks	no	-	a

Shashidhar et al.(24)	2017	India	52/52	54.80%	1223	31	27/38	0	Bifidobacterium longum, Lactobacillus acidophilus, Lactobacillus rhamnosus, Saccharomyces boulardii	1.25×10^9	4 weeks	no	breast milk	a, b, d
Qiao et al.(22)	2017	China	30/30	55.00%	1593	32	-/-	0	Bifidobacterium longum, Lactobacillus acidophilus and Enterococcus faecalis	$1*10^7$	2 weeks	yes	breast milk	a, c
Agus et al.(15)	2011	Indonesia	199/194	48.30%	-	-	-/-	12	Bifidobacterium longum BL999, Lactobacillus rhamonosus LPR	3×10^7	64 weeks	no	breast milk	a, c, d

ALONA et al.(13)	2005	America	72/73	44.10%	1131	30	56/57	0	Bifidobacteria longum infantis, Streptococcus thermophilus, Bifidobacteria bifidus	1.05×10^9	6 weeks	yes	breast milk	b
Hung-Chih et al.(18)	2005	China	180/187	49.90%	1087	28	104/100	0	Bifidobacterium longum infantis, Lactobacillus acidophilus	-	-	no	breast milk	b
Jacobs et al.(7)	2013	Australia	548/551	52.00%	1055	28	359/377	0	Bifidobacterium longum infantis (BB–02), Streptococcus thermophilus (TH–4), Bifidobacterium lactis (BB-12)	1×10^9	-	no	breast milk	b

Carole et al.(23)	2009	France	45/49	42.60%	1085	28	28/35	0	Bifidobacterium longum BB536, Lactobacillus rhamnosus GG	1×10^{8}	2 weeks	yes	breast milk and/or preterm formula	b, c
Bajorek et al.(12)	2021	America	15/15	60.00%	1468	31	10/13	0	Bifidobacterium longum infantis EVC001	8×10^{9}	4 weeks	yes	breast milk	c, d
Jean et al.(14)	2008	France	174/53	51.10%	3400	40	49/19	0	Bifidobacterium longum BL999, Lactobacillus paracasei ST11, Lactobacillus rhamnosus LPR	1.29×10^{8}	16 weeks	no	-	c
Jean-Michel et al.(16)	2011	France	40/39	48.10%	3300	39	3/3	0	Bifidobacterium longum (BL999)	2×10^{7}	16 weeks	no	preterm formula	c

Manzano et al.(19)	2017	Spain	48/51	51.50%	-	-	8/9	6	Bifidobacterium longum infantis R0033	3×10^9	8 weeks	no	breast milk and/or preterm formula	c
Giuseppe et al.(21)	2007	Italy	42/55	53.60%	-	39	15/24	0	Bifidobacterium longum BL999	2×10^7	16 weeks	no	preterm formula	c, d
Stephane et al.(17)	2015	France	145/52	48.70%	1170	29	115/39	0	Bifidobacterium longum, Bifidobacterium lactis	1×10^9	3 weeks	yes	breast milk and/or preterm formula	b, c, d

[1] The left side of the slash is the intervention group and the right side is the control group
[2] a, weight gain; b, necrotizing enterocolitis; c, any adverse events; d, serious adverse events

Table S3 Detailed description of the risk of bias assessment for each included study

Study	Bias	Authors' judgement	Support for judgement
Al-Hosni et al.(6)	bias arising from the randomization process	Unclear risk	"randomised", no details reported.
	bias due to deviations from intended interventions	Low risk	Participants were not aware of their assigned intervention during the trial.
	bias due to missing outcome data	Low risk	Equal and low dropout rates; ITT analysis used.
	bias in the measurement of the outcome	Unclear risk	No information about if outcome assessors aware of the intervention.
	bias in selection of the reported results	Unclear risk	No protocol available.
Mark et al.(20)	bias arising from the randomization process	Low risk	Randomly assigned by the UC Davis Investigational Pharmacy based on a computer generated list; Allocation concealment.
	bias due to deviations from intended interventions	Low risk	Double-blind.
	bias due to missing outcome data	Low risk	Equal dropout rates; ITT analysis used.
	bias in the measurement of the outcome	Unclear risk	No information about if the method of measuring the outcome inappropriate.
	bias in selection of the reported results	Unclear risk	No protocol available.
Shashidhar et al.(24)	bias arising from the randomization process	Unclear risk	No statistical test results for baseline information.
	bias due to deviations from intended interventions	Low risk	Double-blind.
	bias due to missing outcome data	Low risk	Equal and low dropout rates; ITT analysis used.
	bias in the measurement of the outcome	Low risk	Method of measuring the outcome appropriate; Double-blind.
	bias in selection of the reported results	Low risk	Data that produced this result analysed in accordance with a pre-specified analysis plan.
Qiao et al.(22)	bias arising from the randomization process	Low risk	Computer-generated random numbers was used in the sequence generation proces

Study	Bias	Authors' judgement	Support for judgement
	bias due to deviations from intended interventions	Low risk	Double-blind.
	bias due to missing outcome data	Low risk	Data for this outcome available for all, or nearly all, participants randomized.
	bias in the measurement of the outcome	Low risk	Appropriate outcome measures and no intergroup differences; double-blind.
	bias in selection of the reported results	Low risk	Data that produced this result analysed in accordance with a pre-specified analysis plan.
Agus et al.(15)	bias arising from the randomization process	Low risk	Computer-generated random numbers was used in the sequence generation proces
	bias due to deviations from intended interventions	Low risk	Double-blind.
	bias due to missing outcome data	Low risk	Equal and low dropout rates; ITT analysis used.
	bias in the measurement of the outcome	Low risk	Appropriate outcome measures and no intergroup differences; double-blind.
	bias in selection of the reported results	Low risk	Data that produced this result analysed in accordance with a pre-specified analysis plan.
ALON A et al.(13)	bias arising from the randomization process	Unclear risk	"randomised", no details reported.
	bias due to deviations from intended interventions	Low risk	Double-blind; mITT analysis used.
	bias due to missing outcome data	Low risk	Equal and low dropout rates.
	bias in the measurement of the outcome	Low risk	Appropriate outcome measures and no intergroup differences; double-blind.
	bias in selection of the reported results	Unclear risk	No protocol available.
Hung-Chih et al.(18)	bias arising from the randomization process	Low risk	Patients were randomized into the study or control groups by a random-number table sequence.
	bias due to deviations from intended interventions	Low risk	Double-blind; ITT analysis used.
	bias due to missing outcome data	Low risk	Equal and low dropout rates.

Study	Bias	Authors' judgement	Support for judgement
	bias in the measurement of the outcome	Low risk	Appropriate outcome measures and no intergroup differences; double-blind.
	bias in selection of the reported results	Unclear risk	No protocol available.
Jacobs et al.(7)	bias arising from the randomization process	Low risk	Detailed randomization.
	bias due to deviations from intended interventions	Low risk	Double-blind.
	bias due to missing outcome data	Low risk	Equal and low dropout rates.
	bias in the measurement of the outcome	Low risk	Appropriate outcome measures and no intergroup differences; double-blind.
	bias in selection of the reported results	Low risk	Data that produced this result analysed in accordance with a pre-specified analysis plan.
Carole et al.(23)	bias arising from the randomization process	Low risk	Detailed randomization.
	bias due to deviations from intended interventions	Low risk	Double-blind.
	bias due to missing outcome data	Low risk	Equal and low dropout rates; ITT analysis used.
	bias in the measurement of the outcome	Low risk	Appropriate outcome measures and no intergroup differences; double-blind.
	bias in selection of the reported results	Low risk	Data that produced this result analysed in accordance with a pre-specified analysis plan.
Bajorek et al.(12)	bias arising from the randomization process	High risk	Not randomized; no grouping concealment;
	bias due to deviations from intended interventions	High risk	Open-label; no mention about ITT analyis.
	bias due to missing outcome data	Low risk	Equal and low dropout rates.
	bias in the measurement of the outcome	High risk	Open-label.
	bias in selection of the reported results	Unclear risk	No protocol available.

Study	Bias	Authors' judgement	Support for judgement
Jean et al.(14)	bias arising from the randomization process	Low risk	Detailed randomization.
	bias due to deviations from intended interventions	Low risk	Double-blind.
	bias due to missing outcome data	Low risk	Equal and low dropout rates; ITT analysis used.
	bias in the measurement of the outcome	Low risk	Appropriate outcome measures and no intergroup differences; double-blind.
	bias in selection of the reported results	Low risk	Data that produced this result analysed in accordance with a pre-specified analysis plan.
Jean-Michel et al.(16)	bias arising from the randomization process	Low risk	Detailed randomization.
	bias due to deviations from intended interventions	Low risk	Double-blind.
	bias due to missing outcome data	Low risk	Equal and low dropout rates; ITT analysis used.
	bias in the measurement of the outcome	Low risk	Appropriate outcome measures and no intergroup differences; double-blind.
	bias in selection of the reported results	Low risk	Data that produced this result analysed in accordance with a pre-specified analysis plan.
Manzano et al.(19)	bias arising from the randomization process	Low risk	Detailed randomization.
	bias due to deviations from intended interventions	High risk	Using per-protocol analysis.
	bias due to missing outcome data	Low risk	Equal and low dropout rates.
	bias in the measurement of the outcome	Low risk	Appropriate outcome measures and no intergroup differences; double-blind.
	bias in selection of the reported results	High risk	Multiple eligible analyses of the data.
Giuseppe et al.(21)	bias arising from the randomization process	Low risk	Detailed randomization.
	bias due to deviations from intended interventions	Low risk	Double-blind.

Study	Bias	Authors' judgement	Support for judgement
	bias due to missing outcome data	Low risk	Equal and low dropout rates; ITT analysis used.
	bias in the measurement of the outcome	Low risk	Appropriate outcome measures and no intergroup differences; double-blind.
	bias in selection of the reported results	Low risk	Data that produced this result analysed in accordance with a pre-specified analysis plan.
Stephane et al.(17)	bias arising from the randomization process	Low risk	Infants were assigned to their treatment groups according to a pre-established randomization list
	bias due to deviations from intended interventions	Low risk	Double-blind.
	bias due to missing outcome data	Low risk	Equal and low dropout rates; ITT analysis used.
	bias in the measurement of the outcome	Low risk	Appropriate outcome measures and no intergroup differences; double-blind.
	bias in selection of the reported results	Unclear risk	No protocol available.

Table S4 Detailed description of the adverse events for each included study

Study	Publication year	Outcomes[1]	Any adverse events	Serious adverse events
Shashidhar et al.(24)	2017	a, b, d	-	death (1 vs. 3)
Qiao et al.(22)	2017	a, c	Feeding (5 vs. 14) intolerance	-
Agus et al.(15)	2011	a, c, d	Rhinitis<br>Upper respiratory tract infection<br>Diarrhea<br>Fever<br>Coughing Stomatitis<br>Conjunctivitis Vomiting<br>Furunculosis Dermatitis, etc.	2: typhoid fever and dengue encephalopathy<br>4: typhoid fever; fever and febrile seizures; fever, diarrhea, and dehydration; fever, icteric and alcoholic stool, and hepatitis
Carole et al.(23)	2009	b, c	Nosocomial infections (21 vs. 26)	-
Bajorek et al.(12)	2021	c, d	-	2 deaths in control group
Jean et al.(14)	2008	c	Gastroenteritis<br>Gastroesophageal reflux disease Diarrhea<br>Milk allergy Vomiting<br>Febrile infection Surgery<br>Pyrexia<br>Rectal hemorrhage<br>Pyelonephritis<br>Bronchiolitis Cough<br>Drug toxicity Inguinal hernia	
Jean-Michel et al.(16)	2011	c	Gastrointestinal Upper respiratory<br>Lower respiratory<br>Allergy-skin problems<br>urinary tract infection, moniliasis, conjunctivitis, and fever	

Manzano et al.(19)	2017	c	Gastrointestinal disorder<br>Infections and infestations<br>Bone and joint injuries<br>Nervous system disorders<br>Respiratory,thoracic and mediastinal disorders<br>Skin and subcutaneous tissue disorders<br>Eye disorders<br>Ear and labyrinth disorders<br>Fever(LLT)	
Giuseppe et al.(21)	2007	c, d	respiratory tract system (bronchiolitis and cough), etc.	death, etc.
Stephane et al.(17)	2015	b, c, d	no details	death, etc.

[1] a, weight gain; b, necrotizing enterocolitis; c, any adverse events; d, serious adverse events

2.1 Supplementary Figures

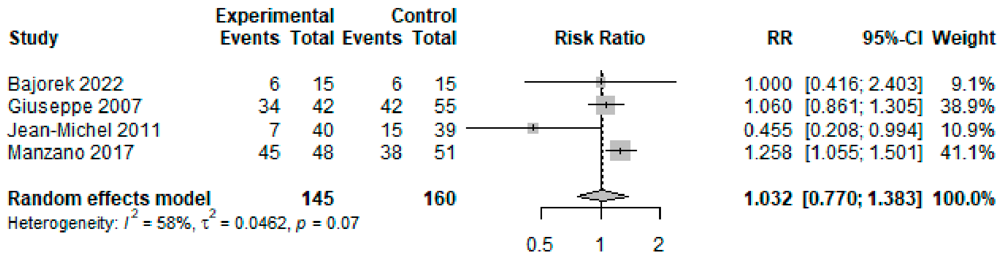

Figure S1. Forest plot comparing the rates of any adverse events for intervention group versus control group in sensitivity analyses on studies containing only Bifidobacterium longum and its subspecies as an intervention group. CI, Confidence interval; RR, risk ratio.

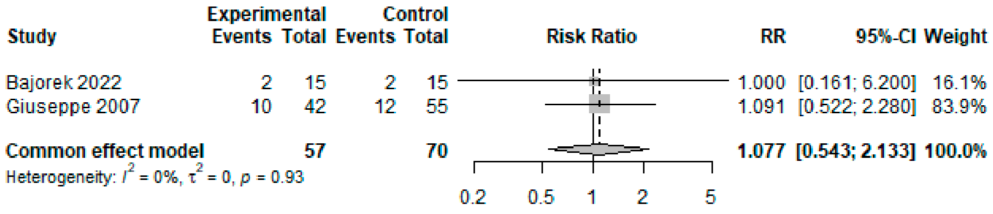

Figure S2. Forest plot comparing the rates of severe adverse events for intervention group versus control group in sensitivity analyses on studies containing only Bifidobacterium longum and its subspecies as an intervention group. CI, Confidence interval; RR, risk ratio

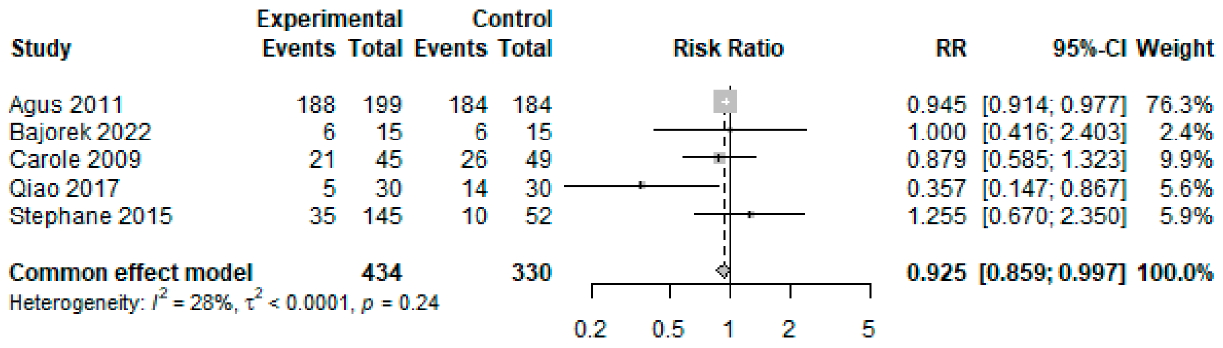

Figure S3. Forest plot comparing the rates of any adverse events for intervention group versus control group in sensitivity analyses among infants below 37-week gestation. CI, Confidence interval; RR, risk ratio

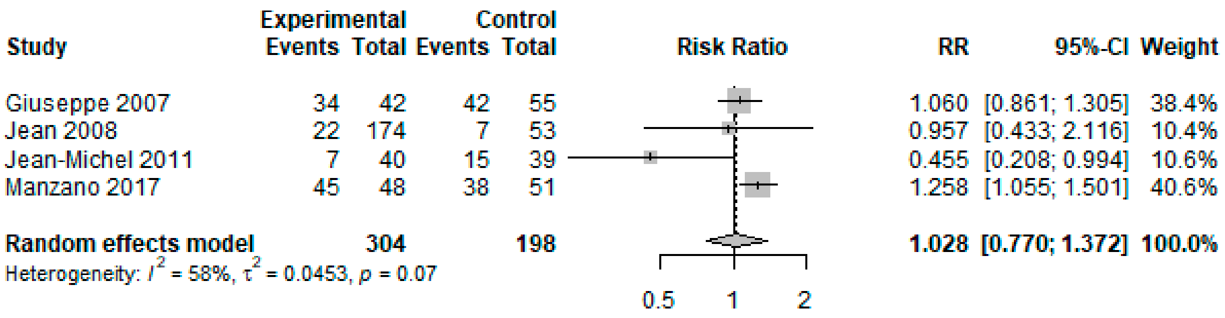

Figure S4. Forest plot comparing the rates of severe adverse events for intervention group versus control group in sensitivity analyses among infants at or above 37-week gestation. CI, Confidence interval; RR, risk ratio

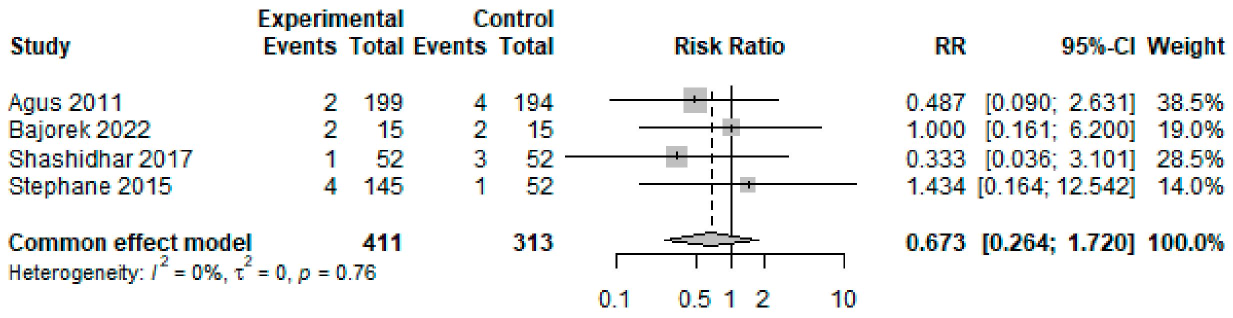

Figure S5. Forest plot comparing the rates of severe adverse events for intervention group versus control group in sensitivity analyses among infants below 37-week gestation. CI, Confidence interval; RR, risk ratio
